# Metabolic reprogramming is associated with symptomatic COVID-19: a serum proteomics and causal inference study identifying ALDOB and glycerol as candidate metabolic correlates

**DOI:** 10.3389/fmicb.2026.1852103

**Published:** 2026-07-24

**Authors:** Nana Guo, Xianlei Zhou, Ziyi Pang, Yan Li, Caixiao Jiang, Minghao Geng, Wentao Wu, Xu Han, Qi Li

**Affiliations:** 1 Hebei Provincial Center for Disease Control and Prevention, Shijiazhuang, Hebei, China; 2 School of Public Health, North China University of Science and Technology, Tangshan, Hebei, China; 3 School of Public Health, Hebei University, Baoding, Hebei, China

**Keywords:** ALDOB, clinical heterogeneity, COVID-19, glycerol, Mendelian randomization, metabolic reprogramming, serum proteomics

## Abstract

**Introduction:**

Coronavirus disease 2019 (COVID-19) shows prominent clinical heterogeneity, presenting two distinct clinical phenotypes including asymptomatic infection and severe symptomatic disease, yet the molecular mechanisms driving such phenotypic differences remain unclear. This study aimed to dissect the molecular basis of divergent COVID-19 clinical manifestations by combining serum proteomics and Mendelian randomization causal inference methods.

**Methods:**

Serum samples from healthy controls, asymptomatic patients (acute and recovery phases), and symptomatic patients (incubation, acute, recovery phases) were subjected to label-free quantitative proteomics to screen stage-specific protein signatures. Functional enrichment analysis was conducted to mine critical biological pathways. Two MR strategies, two-sample MR and SMR, were utilized to infer causal associations between circulating metabolic proteins/traits and COVID-19 susceptibility. This retrospective research did not involve clinical trials (Clinical trial number: Not applicable).

**Results:**

In total, 662 quantifiable serum proteins were detected. Principal component analysis revealed partial separation but obvious overlap across clinical subgroups. Asymptomatic patients only exhibited mild dysregulation of coagulation and innate immune-related proteins, whereas symptomatic patients displayed widespread disorders in coagulation, immunity, metabolism and tissue homeostasis. Five conserved hypoxia and metabolic regulatory proteins (ALDOA, ALDOB, LDHA, LDHB, TFRC) were differentially expressed in both phenotypes. Glycolysis and HIF-1 signaling pathways were disturbed in both groups, with more severe protein expression changes in symptomatic individuals. SMR analysis detected a nominally significant association between ALDOB cis-eQTL variants and COVID-19 risk (*OR* = 1.249, 95% CI: 1.030–1.513, *P* = 0.024). Two-sample MR indicated that higher circulating glycerol was weakly associated with COVID-19 infection (*OR* = 1.14, 95% CI: 1.01–1.28, *P* = 0.027), while global glucose homeostasis traits showed no significant causal links. All MR results were stable after heterogeneity and pleiotropy sensitivity tests.

**Discussion:**

Varied severity of host metabolic-immune dysregulation accounts for different COVID-19 clinical phenotypes. Asymptomatic carriers only experience mild coagulation-immune and metabolic disturbances, whereas symptomatic patients suffer substantially aggravated dysfunction of core metabolic pathways, which provides proteomic and causal evidence for the heterogeneous clinical manifestations of COVID-19.

## 1 Introduction

The global pandemic of coronavirus disease 2019 (COVID-19), caused by severe acute respiratory syndrome coronavirus 2 (SARS-CoV-2), has revealed striking clinical heterogeneity (Lai et al., 2020). A significant proportion of infections remain asymptomatic, with individuals testing positive for viral nucleic acid but lacking recognizable symptoms or radiological abnormalities, while others progress to mild, moderate, or life-threatening severe disease characterized by acute respiratory distress syndrome, systemic inflammation, and multi-organ dysfunction (Wu and McGoogan, 2020; Wei et al., 2020). This variability in clinical outcomes poses substantial challenges to public health surveillance, risk stratification, and targeted intervention strategies. Despite extensive research into the virological and immunological correlates of COVID-19 severity, the molecular mechanisms that distinguish asymptomatic carriage from symptomatic progression remain incompletely defined (Silva et al., 2022; Paes Leme et al., 2024).

Early studies highlighted the role of host immune responses in shaping COVID-19 outcomes, with dysregulated innate immunity, delayed type I interferon production, and hyperactive adaptive immune responses linked to severe disease (Blanco-Melo et al., 2020; Liu et al., 2025). However, these findings do not fully explain the absence of symptoms in asymptomatic individuals, who often mount detectable immune responses yet avoid systemic inflammation (Chen et al., 2020). More recent work has shifted focus to systemic molecular signatures, including proteomic and metabolomic perturbations, which offer insights into the global biological changes underlying clinical phenotypes (Dutsch et al., 2023; Overmyer et al., 2021; Nie et al., 2021). Serum proteomics, in particular, has emerged as a powerful tool to characterize disease-associated molecular cascades, with studies identifying coagulation dysfunction, complement activation, and metabolic reprogramming as key features of symptomatic COVID-19 (Shen et al., 2020; Sameh et al., 2023). Nevertheless, direct cross-sectional comparative analyses of proteomic profiles across different disease stages in asymptomatic and symptomatic patients are lacking, limiting our understanding of key molecular features linked to different clinical outcomes.

Metabolic dysfunction has emerged as a central theme in COVID-19 pathogenesis. Symptomatic patients exhibit marked perturbations in glycolysis, gluconeogenesis, and lipid metabolism, with a “Warburg-like” shift toward aerobic glycolysis in immune and parenchymal cells (Marchetti, 2020; Codo et al., 2020). Hypoxia-inducible factor 1 (HIF-1) signaling, a key regulator of metabolic adaptation to hypoxia and inflammation, is also dysregulated in severe disease, linking metabolic reprogramming to tissue hypoxia and inflammatory amplification (da Silva et al., 2025; Serebrovska et al., 2020). However, whether such metabolic changes are universal across COVID-19 phenotypes or specific to symptomatic progression remains unclear. Additionally, while observational studies have associated pre-existing metabolic conditions, such as diabetes (Abdi et al., 2020), obesity (Cava et al., 2021), with severe COVID-19, causal relationships between specific metabolic traits and disease susceptibility remain to be established.

Functional genomics approaches, including Mendelian randomization (MR) and Summary Mendelian Randomization (SMR), offer powerful tools to dissect causal relationships between molecular traits and disease outcomes, mitigating confounding and reverse causality inherent in observational studies (Davey Smith and Hemani, 2014). SMR, which integrates expression quantitative trait loci (eQTLs) or protein quantitative trait loci (pQTLs) with genome-wide association study (GWAS) data, enables identification of genes whose expression or protein levels causally influence disease risk (Zhu et al., 2016). Similarly, two-sample MR can elucidate causal effects of metabolic traits on COVID-19 by leveraging genetic variants as instrumental variable (Burgess et al., 2013). Integrating these causal inference methods with proteomic profiling provides a promising strategy to explore key molecular factors driving COVID-19 clinical heterogeneity across different disease stages.

This study integrates serum proteomics and GWAS-based Mendelian randomization analyses to explore the molecular mechanisms underlying diverse COVID-19 clinical phenotypes. We performed label-free serum proteomic profiling on healthy controls and COVID-19 patients divided into detailed clinical stages to characterize stage-specific molecular signatures. Functional enrichment and protein–protein interaction analyses were applied to identify key biological pathways related to different clinical phenotypes. We also screened shared core molecules across groups and adopted SMR and two-sample MR to explore the relationships between metabolic molecules and COVID-19 susceptibility. The results offer a reference framework for interpreting COVID-19 clinical heterogeneity and uncover candidate metabolic molecules for subsequent risk stratification and intervention research.

Collectively, current COVID-19 omics studies have two prominent limitations: first, most proteomic analyses fail to stratify patients into refined disease stages, lacking systematic comparisons of stage-specific molecular features between asymptomatic and symptomatic populations; second, existing observational proteomic findings cannot distinguish correlation from causation, and few studies combine staged proteomic data with multiple Mendelian randomization approaches to validate phenotype-related metabolic molecules. To fill these research gaps, the present study performed label-free serum proteomics on healthy controls and COVID-19 patients grouped by detailed clinical stages. We further combined SMR and two-sample MR analyses to explore associations between core metabolic molecules and COVID-19 susceptibility. This multi-omics and causal inference integrated design distinguishes our work from previous single-group or single-omics studies, and the results may provide new clues for explaining the clinical heterogeneity of COVID-19.

## 2 Materials and methods

### 2.1 Study participants and sample collection

This study was approved by the Ethics Committee of the Hebei Provincial Centre for Disease Control and Prevention (Approval No.: HeBIRBS2024020), and written informed consent was obtained from all participants. Serum samples were derived from a regional cluster outbreak of COVID-19 that occurred in 2021, which were collected during the official investigation of outbreak prevention and control measures and epidemiological characteristics. This study included a total of 40 samples. There were 12 samples from asymptomatic infections (ASY), including 6 from the asymptomatic phase and 6 from the recovery phase; 21 samples from confirmed cases, including 7 from the incubation period, 8 from the clinical phase, and 6 from the recovery phase; and 7 samples from healthy individuals. There were no statistically significant differences in age or gender among the groups (*P* > 0.05; Supplementary Table 1).

Participants were stratified into three main groups with detailed subgrouping criteria as follows: (1) Healthy Population Group (HEA): Individuals who tested negative for SARS-CoV-2 via nucleic acid detection and showed negative results for both anti-SARS-CoV-2 IgM and IgG antibodies. These individuals had no history of COVID-19 exposure within 14 days prior to sampling and no clinical symptoms suggestive of respiratory infection. (2) Confirmed Symptomatic Cases Group (SYM): Individuals diagnosed as confirmed COVID-19 cases according to the Diagnosis and Treatment Protocol for Novel Coronavirus Pneumonia (Eighth Edition) issued by the National Health Commission of China. This group was further divided into three stages based on disease progression: Incubation period (SYM-1): Samples collected from confirmed cases who tested positive for SARS-CoV-2 nucleic acid but had not yet developed any clinical symptoms or signs (fever, cough, fatigue, myalgia, sore throat, dyspnea). Clinical symptom period (SYM-2): Samples collected from confirmed cases during the phase when they presented with typical clinical symptoms and signs of COVID-19, with symptom duration ranging from 1 to 14 days. Recovery period (SYM-3): Samples collected from confirmed cases who had met the discharge criteria, including resolution of clinical symptoms, negative SARS-CoV-2 nucleic acid test results for two consecutive times (sampling interval ≥24 h), and normal chest computed tomography (CT) findings. (3) Asymptomatic Infection Group (ASY): Individuals who tested positive for SARS-CoV-2 nucleic acid but had no clinically recognizable symptoms or signs throughout the entire observation period, and chest CT scans showed no radiological features of COVID-19 (ground-glass opacities, consolidation). All individuals in this group completed a 14-day isolated medical observation and remained asymptomatic during this period. This group was subdivided into two stages: Asymptomatic infection period (ASY-1): Samples collected during the phase when individuals tested positive for SARS-CoV-2 nucleic acid. Asymptomatic recovery period (ASY-2): Samples collected after individuals converted to negative for SARS-CoV-2 nucleic acid following the 14-day isolated medical observation. Serum samples (5 ml per participant) were collected via venous blood draw, centrifuged at 3,000 × g for 15 min at 4 °C to separate serum, and immediately stored at −80 °C until further analysis to avoid repeated freeze-thaw cycles.

### 2.2 Serum proteomic profiling

#### 2.2.1 Protein extraction and digestion

Serum proteins were extracted using the urea lysis method. Briefly, 100 μl of serum was mixed with 400 μl of lysis buffer (8 M urea, 100 mM Tris-HCl, pH = 8.0, containing 1% protease inhibitor cocktail and 1% phosphatase inhibitor cocktail). The mixture was sonicated on ice for 30 s (3 cycles, 10 s on/20 s off) and centrifuged at 12,000 × g for 20 min at 4 °C to remove insoluble debris. The protein concentration in the supernatant was determined using the BCA Protein Assay Kit (Thermo Fisher Scientific, Waltham, MA, USA) according to the manufacturer's instructions.

For protein digestion, 100 μg of total protein from each sample was reduced with 10 mM dithiothreitol (DTT) at 56 °C for 30 min, then alkylated with 20 mM iodoacetamide (IAA) in the dark at room temperature for 30 min. The alkylated protein solution was diluted 4-fold with 50 mM Tris-HCl (pH = 8.0) to reduce urea concentration to ≤ 2 M, and trypsin (Promega, Madison, WI, USA) was added at a protein-to-trypsin ratio of 50:1. Digestion was performed at 37 °C for 16 h, and the reaction was terminated by adding formic acid (final concentration 1%). The digested peptides were desalted using C18 solid-phase extraction columns (Thermo Fisher Scientific, Waltham, MA, USA), dried via vacuum centrifugation, and resuspended in 0.1% formic acid for LC-MS/MS analysis.

#### 2.2.2 LC-MS/MS analysis

Prior to LC-MS analysis, high-abundance serum proteins including albumin and immunoglobulins were depleted using the ProteoExtract™ Albumin/IgG Removal Kit (Merck KGaA, Darmstadt, Germany) according to the manufacturer's instructions. Briefly, thawed serum samples were diluted with dilution buffer, loaded onto the pre-equilibrated spin column, and incubated at room temperature to facilitate specific binding of albumin and IgG. After brief centrifugation, the flow-through fractions containing the remaining serum proteins were collected, then concentrated and desalted using Amicon Ultra centrifugal filter units prior to LC-MS analysis. Peptide separation was performed using an Ultimate 3000 RSL Cnano system (Thermo Fisher Scientific) equipped with a C18 trap column (Acclaim PepMap 100, 75 μm × 2 cm, 3 μm, 100 A) and an analytical column (Acclaim PepMap RSLC, 75 μm × 15 cm, 2 μm, 100 A). The mobile phase consisted of solvent A (0.1% formic acid in water) and solvent B (0.1% formic acid in acetonitrile). Peptides were loaded onto the trap column at a flow rate of 5 μl/min with 2% solvent B for 5 min, then eluted onto the analytical column with a linear gradient of solvent B from 2% to 35% over 90 min, followed by a gradient from 35% to 90% solvent B for 5 min, and held at 90% solvent B for 5 min. The column temperature was maintained at 40 °C, and the flow rate was set to 300 nl/min.

MS/MS analysis was performed on a Q Exactive Plus mass spectrometer (Thermo Fisher Scientific, Waltham, MA, USA) coupled with the LC system via a nano-electrospray ionization source. The mass spectrometer was operated in positive ion mode with a spray voltage of 2.1 kV and a capillary temperature of 320 °C. Full MS scans were acquired in the range of m/z 350–1,800 with a resolution of 70,000 (at m/z 200), an automatic gain control (AGC) target of 3e6, and a maximum injection time of 50 ms. The top 10 most abundant precursor ions from each full MS scan were selected for MS/MS fragmentation via higher-energy collisional dissociation (HCD) with a normalized collision energy of 27%. MS/MS scans were acquired with a resolution of 17,500 (at m/z 200), an AGC target of 1e5, a maximum injection time of 60 ms, and an isolation window of 1.6 m/z. Dynamic exclusion was set to 30 s to avoid repeated fragmentation of the same precursor ion.

### 2.3 Bioinformatics analysis

#### 2.3.1 Proteomic data preprocessing

Raw LC-MS/MS data were processed using MaxQuant software (version 1.6.17.0) for peptide identification and protein quantification. The search was performed against the UniProt Human Reference Proteome database (UP000005640, released in 2021) containing 20,329 protein entries, with the addition of a common contaminants database. The search parameters were set as follows: enzyme specificity was trypsin with cleavage after lysine (K) or arginine (R) residues, excluding proline (P) at the cleavage site; mass tolerance for precursor ions was 20 ppm for the first search and 5 ppm for the main search; mass tolerance for fragment ions was 0.5 Da; carbamidomethylation of cysteine was set as a fixed modification; oxidation of methionine and acetylation of protein N-termini were set as variable modifications. Label-free quantification (LFQ) was enabled with a minimum ratio count of 2. After database searching, the resulting protein groups were filtered to remove contaminants, reverse hits, and proteins identified by fewer than 2 unique peptides. The remaining protein intensity data were imported into R software (version 4.2.2) for further analysis. Prior to downstream analysis, the LFQ intensity values extracted from MaxQuant were used for subsequent bioinformatics analysis instead of raw intensity values. All LFQ intensity values were log2-transformed to stabilize variance, and median centering normalization was implemented by the normalizeQuantiles function embedded in the limma package (version 3.52.4) to eliminate systematic technical biases across samples (Ritchie et al., 2015). The false discovery rate (FDR) was set to 0.01 at the peptide-spectrum match (PSM), peptide, and protein levels for identification.

#### 2.3.2 Principal component analysis (PCA)

To assess global sample similarity and separation, PCA was conducted using the log2-transformed and normalized protein intensity matrix. First, to reduce background noise, proteins identified and detected in fewer than 80% of all samples were excluded at the raw identification level as a preliminary screening step. After this pre-filtering, data normalization and log2 transformation were performed to construct the quantitative matrix. Subsequently, only proteins with 100% complete and non-missing quantitative intensity profiles across all remaining samples were retained for PCA and downstream analyses; no missing value imputation was performed in this study. A total of 87 proteins were removed due to incomplete quantitative profiles. These excluded proteins were mainly low-abundance serum proteins, and no systematic differences in missing patterns or abundance distributions were observed across different clinical groups. The PCA was performed using the prcomp function in the R base stats package, with the input data auto-scaled to ensure that proteins with different intensity ranges contributed equally to the analysis.

#### 2.3.3 Differential protein expression analysis

Differentially expressed proteins (DEPs) between pairwise comparison groups (HEA vs. SYM-1, HEA vs. SYM-2, HEA vs. SYM-3, HEA vs. ASY-1, HEA vs. ASY-2) were identified using the “limma” package in R. Linear models were fitted to the log2-transformed protein intensity values, with the group factor as the main variable. Empirical Bayes moderation was applied to shrink the standard errors of the estimated coefficients, improving the statistical power for small sample sizes. The median technical coefficient of variation (CV) for all quantified proteins across repeated quality control samples was 11.3%, indicating reliable technical reproducibility of our proteomic platform. The FDR was used for multiple-testing correction. DEPs were defined with the following thresholds: adjusted *P*-value (FDR) < 0.05 and absolute fold change ≥1.2. This fold-change threshold was selected according to the platform's median technical CV: a cut-off of 1.2 can effectively separate true biological expression differences from technical noise, and it is a commonly adopted criterion for clinical serum proteomic studies to retain biologically relevant proteins with moderate expression changes.

#### 2.3.4 Functional enrichment analysis

Functional annotation and over-representation analysis (ORA) of DEPs were performed using the clusterProfiler package (version 4.4.4) in R (Yu et al., 2012). Gene Ontology (GO) enrichment analysis included three categories: Biological Process (BP), Cellular Component (CC), and Molecular Function (MF). Kyoto Encyclopedia of Genes and Genomes (KEGG) pathway enrichment analysis was also conducted to identify significantly enriched signaling pathways (Kanehisa and Goto, 2000). ORA was performed separately for up-regulated DEPs, down-regulated DEPs, and the total combined DEPs for independent functional interpretation. To ensure analytical rigor, the background gene set for all enrichment analyses was strictly defined as all proteins quantified in this study, instead of the whole human proteome, which minimizes background interference. The statistical significance of enrichment was determined using the hypergeometric test, and the resulting *P*-values were adjusted by the FDR correction method with a significance threshold of *FDR* < 0.05.

#### 2.3.5 Protein–protein interaction (PPI) network construction and analysis

PPI networks of DEPs were constructed using the STRING database (version 11.5), which integrates known and predicted functional associations as well as physical protein interactions from multiple sources (Szklarczyk et al., 2021). The network herein reflects comprehensive functional association relationships rather than only direct physical interactions. The confidence score threshold was set to ≥0.7 (high confidence) to ensure the reliability of the network.

#### 2.3.6 Statistical analysis and visualization

All statistical tests were two-sided, and *P* < 0.05 were considered statistically significant unless otherwise specified. Continuous data were presented as median (interquartile range, IQR) due to non-normal distribution, and differences between groups were compared using the Wilcoxon rank-sum test. Visualizations including volcano plots, bar plots, bubble plots, Venn diagrams, and boxplots were generated using the “enhanced Volcano”, “ggplot2”, “vennDiagram”, and “pheatmap” (Li et al., 2023; Wu et al., 2021; Chen and Boutros, 2011; Hu, 2021) packages in R. Hierarchical clustering for heatmap generation was performed using Euclidean distance and complete linkage method, with rows (proteins) and columns (samples) scaled to *Z*-scores.

### 2.4 Mendelian randomization analysis

#### 2.4.1 Summary-based Mendelian randomization study (SMR)

SMR analysis and the heterogeneity in dependent instruments (HEIDI) method possess the capability to distinguish between multi-effect models and linkage models (Zhu et al., 2016). GWAS Data Sources: GWAS data for 4,907 cis-protein quantitative trait loci (cis-pQTLs) were sourced from the deCODE Genetics database (Ferkingstad et al., 2021), encompassing plasma pQTL information from 35,559 Icelandic individuals. Cis-expression quantitative trait loci (cis-eQTLs) data were derived from the eQTLGen Consortium database, comprising blood samples from 31,684 European individual (Vosa et al., 2021). The COVID-19 GWAS dataset utilized was sourced from the FinnGen R12 project, comprising suspected cases (9,587 cases and 47,886 controls) and confirmed cases (12,478 cases and 47,886 controls). Perform SMR analysis using SMR software (v1.3.1) to confirm causal relationships between exposure and outcomes. The HEIDI test examines whether potential pleiotropy and linkage disequilibrium exist between exposure and outcomes. When *P*_SMR_ < 0.05 and *P*_HEIDI_ > 0.05, the causal association between exposure and outcome is considered driven by shared genetic variation. In this proteome-wide SMR screen covering 4,907 proteins, we did not perform multiple testing correction. This approach is commonly used in large-scale omics-based MR (Yuan et al., 2023; Zhang et al., 2022; Sun et al., 2024; Wang et al., 2024) studies. Strict multiple testing correction tends to exclude nominally significant signals with potential biological functions, and such preliminary clues are retained in our study for subsequent mechanism exploration. It should be noted that results derived from the nominal threshold carry a risk of false positives, and all screened candidates require further independent validation. Notably, all cis-pQTL and cis-eQTL datasets used as genetic instrumental variables (IVs) were generated from European populations, while the serum proteomic samples in the present study were collected from a Chinese cohort. Genetic regulatory effects of eQTLs and pQTLs are well documented to be strongly ancestry-specific: only 30–50% of cis-regulatory effects are shared between European and East Asian populations, due to systematic differences in allele frequencies, linkage disequilibrium structures, and regulatory sequence polymorphisms (Storey et al., 2007; Stranger et al., 2012). This ancestry mismatch may reduce the statistical power of SMR analyses and introduce potential bias in effect size estimation. Findings from this analysis should be interpreted with great caution, and all identified associations require independent validation in East Asian population cohorts. The GWAS summary statistics used for exposure and outcome were derived from completely independent cohorts, with no sample overlap. Therefore, no additional adjustment for overlap was required.

#### 2.4.2 Two-sample Mendelian randomization

Exposure data included clinical indicators of glucose metabolism and eight glucose metabolism intermediate product indicators, all retrieved from the GWAS Catalog database (https://www.ebi.ac.uk/gwas/downloads/summary-statistics); the clinical indicators of glucose metabolism were fasting blood glucose (GCST005186), fasting insulin (GCST90002238), and insulin resistance (GCST005179), and the glucose metabolism intermediate product indicators were citrate level (GCST90132705), glucose level (GCST90019508), glycerol level (GCST90132726), lactate level (GCST90092882), pyruvate level (GCST90132743), glucose-6-phosphate dehydrogenase level (GCST90439055), glutamine-fructose-6-phosphate transaminase 1 level (GCST90440845), and 1,5-anhydroglucitol level (GCST90199648). Mendelian randomization (MR) analysis was performed using R software (version 4.4.2) with the “TwoSampleMR” package (Hemani et al., 2018), where instrumental variables (IVs; single nucleotide polymorphisms, SNPs) must meet three core assumptions: (1) Association Assumption: SNPs are significantly associated with exposure; (2) Independence Assumption: SNPs are independent of potential confounders between exposure and outcome; and (3) Exclusivity Assumption: SNPs influence the outcome solely through exposure (Bowden and Holmes, 2019). To ensure an adequate number of instrumental variables for each exposure, SNPs were selected at *P* < 5 × 10^−6^ when the conventional threshold (*P* < 5 × 10^−8^) yielded too few instruments. SNPs strongly associated with exposure (*P* < 5 × 10^−6^) were selected as IVs, with linkage disequilibrium pruned within a 10,000 kb window using *R*^2^ < 0.001, and SNPs with *F* > 10 retained to exclude weak IV bias. The number of included SNPs for each exposure and their corresponding *F*-statistic values are summarized in the Supplementary Table 2. All instrumental variables had *F*-statistics >10, indicating no evidence of weak instruments. Five statistical methods [MR Egger, weighted median, simple mode, weighted mode, and inverse-variance weighted (IVW)] were used to estimate the exposure-outcome association, with a significant causal relationship defined as IVW *P* < 0.05 (Cao et al., 2023). Robustness was verified via the leave-one-out (de-replication) method; the MR-Egger intercept test was used to assess level-specific horizontal pleiotropy, and Cochrane's *Q*-test was applied to examine potential heterogeneity, with *P* < 0.05 indicating significant pleiotropy or heterogeneity. Finally, reverse MR analysis was conducted to evaluate potential reverse causality. All GWAS summary statistics for both metabolic exposures and COVID-19 outcomes in this two-sample MR analysis were derived from European ancestry populations. Given our Chinese proteomic cohort, inter-population genetic differences may attenuate the effect of selected instrumental variables and limit the generalizability of MR effect estimates to East Asian populations. This inherent limitation should be carefully considered when interpreting the results, and all causal inferences are considered preliminary exploratory findings.

## 3 Results

### 3.1 Serum proteomic profiling reveals distinct molecular signatures across COVID-19 clinical phenotypes

**Figure 1:**
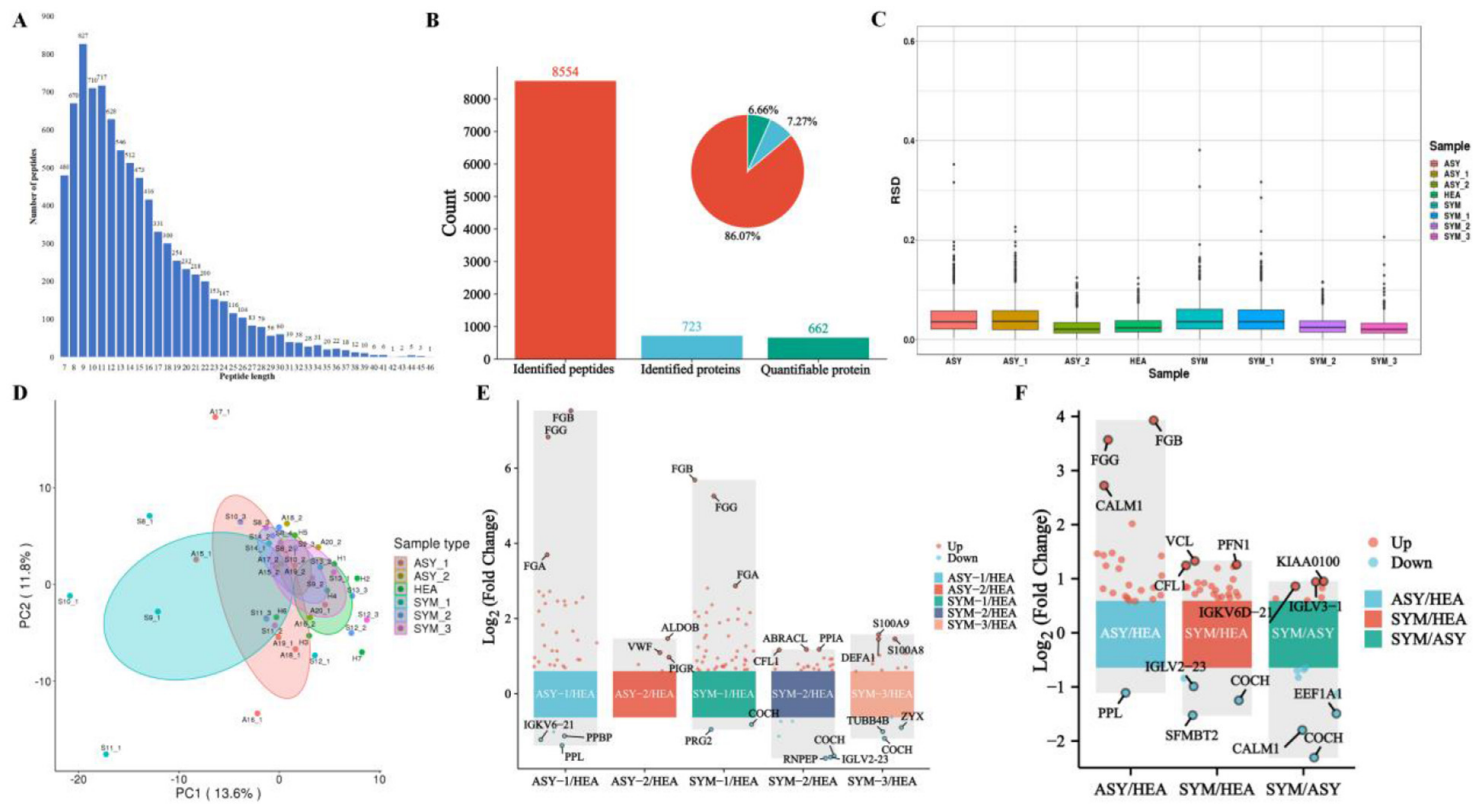
Peptide and protein profiling in samples. **(A)** Distribution of peptide lengths across samples. The *x*-axis denotes peptide length; *y*-axis, peptide count. **(B)** Total identified peptides (8,554), identified proteins (723), and quantifiable proteins (662; pie chart: proportional distribution). **(C)** Relative standard deviation (RSD) of protein quantification across samples (box plots represent RSD ranges). **(D)** PCA of samples (PC1: 13.6%, PC2: 11.8%). Colors/shapes distinguish sample types (ASY, HEA, SYM subgroups). **(E)** Volcano plot of log_2_ (fold change) for protein expression (ASY/HEA, SYM/HEA subgroups). Red/blue dots: up/downregulated proteins; labeled proteins are key candidates. **(F)** Summary of log_2_ (fold change) in three comparisons (ASY/HEA, SYM/HEA, SYM/ASY). Top/bottom proteins (up/downregulated) are labeled. All panels: samples include ASY (asymptomatic), HEA (healthy), SYM (symptomatic) and their subgroups.

To characterize the serum proteome landscape of COVID-19, we performed label-free LC-MS/MS analysis on samples from HEA, asymptomatic infections (ASY; subdivided into ASY-1 and ASY-2 phases), and symptomatic cases (SYM; subdivided into incubation SYM-1, SYM-2, and SYM-3 phases). A total of 8,554 peptides were identified, mapping to 723 proteins (662 of which were quantifiable; Figure 1B), with peptide lengths predominantly distributed between 7 and 20 amino acids (Figure 1A), consistent with tryptic digestion specificity. Technical reproducibility was generally good across all sample groups, as evidenced by the low median relative standard deviation (RSD) in protein quantification (median *RSD* < 0.1). Notably, a small number of outliers with *RSD* > 0.2 were observed, which were predominantly concentrated in the symptomatic acute-phase groups (SYM, SYM-1 and SYM-3). Given the stable technical reproducibility confirmed by QC samples (median *CV* = 11.3%), these elevated RSD values likely reflect the high biological heterogeneity of systemic inflammatory responses in symptomatic patients rather than technical artifacts (Figure 1C). Global proteomic clustering via PCA revealed a partial separation trend between clinical groups, with substantial overlap among most subgroups: healthy control (HEA) samples clustered tightly, while asymptomatic (ASY) and symptomatic (SYM) samples showed only a weak tendency to segregate along PC1 (13.6% variance) and PC2 (11.8% variance). The cumulative variance explained by the first 10 principal components was 72.1%, capturing the majority of the total variability in the dataset. This pattern suggests some degree of molecular profile differences associated with disease status, but also reflects the high biological heterogeneity of COVID-19 patient samples (Figure 1D). This study screened differentially expressed proteins in five pairwise comparison groups (ASY-1/HEA, ASY-2/HEA, SYM-1/HEA, SYM-2/HEA, SYM-3/HEA) and three combined groups (ASY/HEA, SYM/HEA, SYM/ASY; Figures 1E, F). In the individual pairwise comparison analysis (Figure 1E), fibrinogen family proteins FGB, FGG, and FGA were the most significantly upregulated proteins in the ASY-1/HEA and SYM-1/HEA comparison groups, while S100A8, S100A9, and DEFA1 were markedly upregulated in the SYM-3/HEA group. In contrast, COCH protein was consistently downregulated in the SYM-1/HEA, SYM-2/HEA, and SYM-3/HEA groups; the SYM subgroups also showed downregulation of PRG2, RNPEP, IGLV2-23, TUBB4B, and ZYX, whereas the ASY-1/HEA group exhibited downregulation of IGKV6-21, PPBP, and PPL proteins. The combined group analysis (Figure 1F) further confirmed these trends: in the ASY/HEA group, FGB, FGG, and CALM1 were significantly upregulated, with only PPL showing notable downregulation; in the SYM/HEA group, CFL1, VCL, and PFN1 were upregulated, while COCH, SFMBT2, and IGLV2-23 were downregulated; the SYM/ASY direct comparison group displayed a unique pattern of protein expression disturbance, with upregulation of IGLV3-1 and KIAA0100 and downregulation of CALM1, COCH, and EEF1A1. In summary, our data confirm that fibrinogen family proteins are core upregulated differentially expressed proteins in both ASY/HEA and SYM/HEA systems, COCH is a commonly downregulated marker protein across multiple SYM subgroups, and both SYM and ASY treatment systems exhibit their own distinctive immunoglobulin and calcium-binding protein expression features.

### 3.2 Serum proteomic and functional profiling defines a targeted, phase-dependent molecular signature of asymptomatic COVID-19

**Figure 2:**
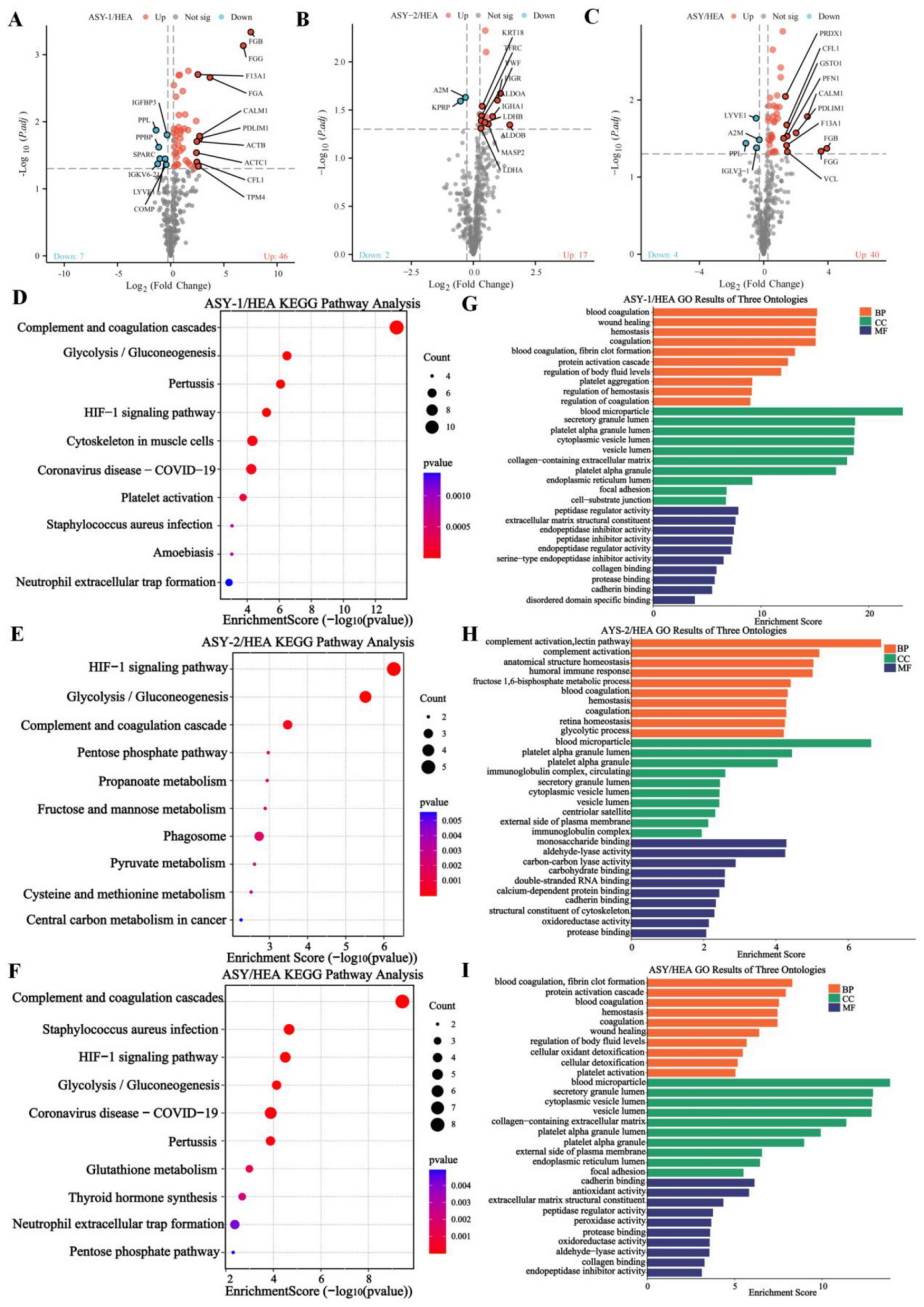
ASY vs. HEA: protein expression and functional enrichment. **(A–C)** Volcano plots of protein expression (ASY-1/HEA, ASY-2/HEA, ASY/HEA). *x*-axis: log_2_ (fold change); *y*-axis: –log_10_ (*P*-value). Dots: proteins (red: upregulated; blue: downregulated; gray: not significant). Labeled proteins are key candidates. **(D**–**F)** KEGG pathway enrichment (ASY-1/HEA, ASY-2/HEA, ASY/HEA). *x*-axis: enrichment score [–log_10_ (*P*-value)]; *y*-axis: pathways. Dot size: gene count; color: *P*-value. **(G–I)** GO term enrichment (BP, biological process; CC, cellular component; MF, molecular function) for ASY-1/HEA, ASY-2/HEA, ASY/HEA. *x*-axis: enrichment score; *y*-axis: terms. Colors distinguish GO categories.

To define the molecular circuitry of asymptomatic COVID-19, we analyzed serum proteomic dysregulation and pathway enrichment across ASY-1 and ASY-2 infections relative to HEA. The volcano plots showed that ASY-1 drives robust upregulation of 46 proteins and these proteins are dominated by coagulation factors (FGA, FGB) and innate immune mediators (CALM1), while only 7 proteins, such as LYVE1, are downregulated (Figure 2A). By ASY-2, this signature contracts: 17 proteins (including metabolic enzymes ALDOA, LDHA) are upregulated and 2 proteins are downregulated, which reflects post-infection recovery (Figure 2B). The combined ASY analysis consolidates these changes, with 40 upregulated proteins (FGA, VCL) and 4 downregulated (LYVE1), which highlights core asymptomatic-specific perturbations (Figure 2C). Notably, the ASY-1 signature prioritizes coagulation and immune effectors. These align with early symptomatic changes but have reduced magnitude. Meanwhile, ASY-2 shifts toward metabolic restoration, which is consistent with the limited inflammatory burden of asymptomatic infection. These cross sectional comparisons reveal distinct molecular features of different asymptomatic disease phases: a focused response signature in the active infection phase and a metabolism oriented profile in the recovery phase. This pattern of constrained, phase specific molecular alterations distinguishes asymptomatic infection from the widespread dysregulation observed in symptomatic COVID-19. Functional annotation via ORA for KEGG pathways further resolved the proteomic dynamics of asymptomatic COVID-19. For ASY-1, enriched pathways included complement and coagulation cascades and coronavirus disease-COVID-19. These pathways appeared alongside HIF-1 signaling and Glycolysis/Gluconeogenesis, which aligns with the upregulation of coagulation and metabolic proteins in this phase (Figure 2D). In the enrichment plots, the *x*-axis represents the enrichment score, defined as the –log_10_-transformed *P*-value, and the color scale also reflects the enrichment significance based on –log_10_ (*P*-value). By ASY-2, 17 proteins (including metabolic enzymes ALDOA, LDHA) are upregulated and 2 proteins are downregulated. Among these 17 upregulated proteins, four core metabolic enzymes (ALDOA, ALDOB, LDHA, LDHB) were annotated to both HIF-1 signaling and glycolysis/gluconeogenesis pathways. In ASY-2, pathway enrichment contracted to a smaller set. This set is dominated by HIF-1 signaling and Glycolysis/Gluconeogenesis (*FDR* < 0.05), which reflects the resolution of acute immune responses and restoration of metabolic homeostasis (Figure 2E). The combined asymptomatic cohort (ASY/HEA) consolidated these signatures. Top enrichment included complement and coagulation cascades and *Staphylococcus aureus* infection (a proxy for innate immune activation), alongside persistent representation of HIF-1 signaling and metabolic pathways (Figure 2F). All pathway and GO enrichment analyses in this section were performed using the pooled set of differentially expressed proteins (both up- and down-regulated proteins), rather than separate analyses for up- and down-regulated subsets. The GO enrichment analysis delineated the BP, CC, and MF underlying asymptomatic COVID-19 proteomic changes. For ASY-1, BP enrichment was dominated by coagulation-related processes, while CC terms prioritized platelet granules and extracellular matrix components. MF signatures centered on protease activity and binding, consistent with the pro-thrombotic proteomic profile of this phase (Figure 2G). In ASY-2, the GO signature shifted: BP terms included complement activation and fructose metabolism, CC terms focused on immune complexes and vesicle lumens, and MF signatures emphasized carbohydrate binding, reflecting the transition from acute pathogen response to metabolic and immune resolution (Figure 2H). The combined ASY cohort consolidated these trends: BP enrichment retained blood coagulation as a top term, CC terms highlighted platelet and extracellular compartments, and MF signatures persisted for protease activity (Figure 2I). Together, these cross sectional proteomic and functional analyses demonstrated that different clinical phases of asymptomatic COVID-19 exhibit distinct molecular profiles: the active infection phase was characterized by enrichment of coagulation and innate immune pathways, while the recovery phase showed predominant metabolic and immune homeostasis related signatures. This targeted, reversible pathway based on thrombus remodeling and confined to the pathogen response process defines asymptomatic infection as a distinct clinical phenotype functionally dissociated from the extensive inflammation and systemic dysregulation characteristic of symptomatic COVID-19.

### 3.3 Serum proteomic and functional profiling reveals stage-specific coagulation, metabolic, and immune dysregulation in symptomatic COVID-19

**Figure 3:**
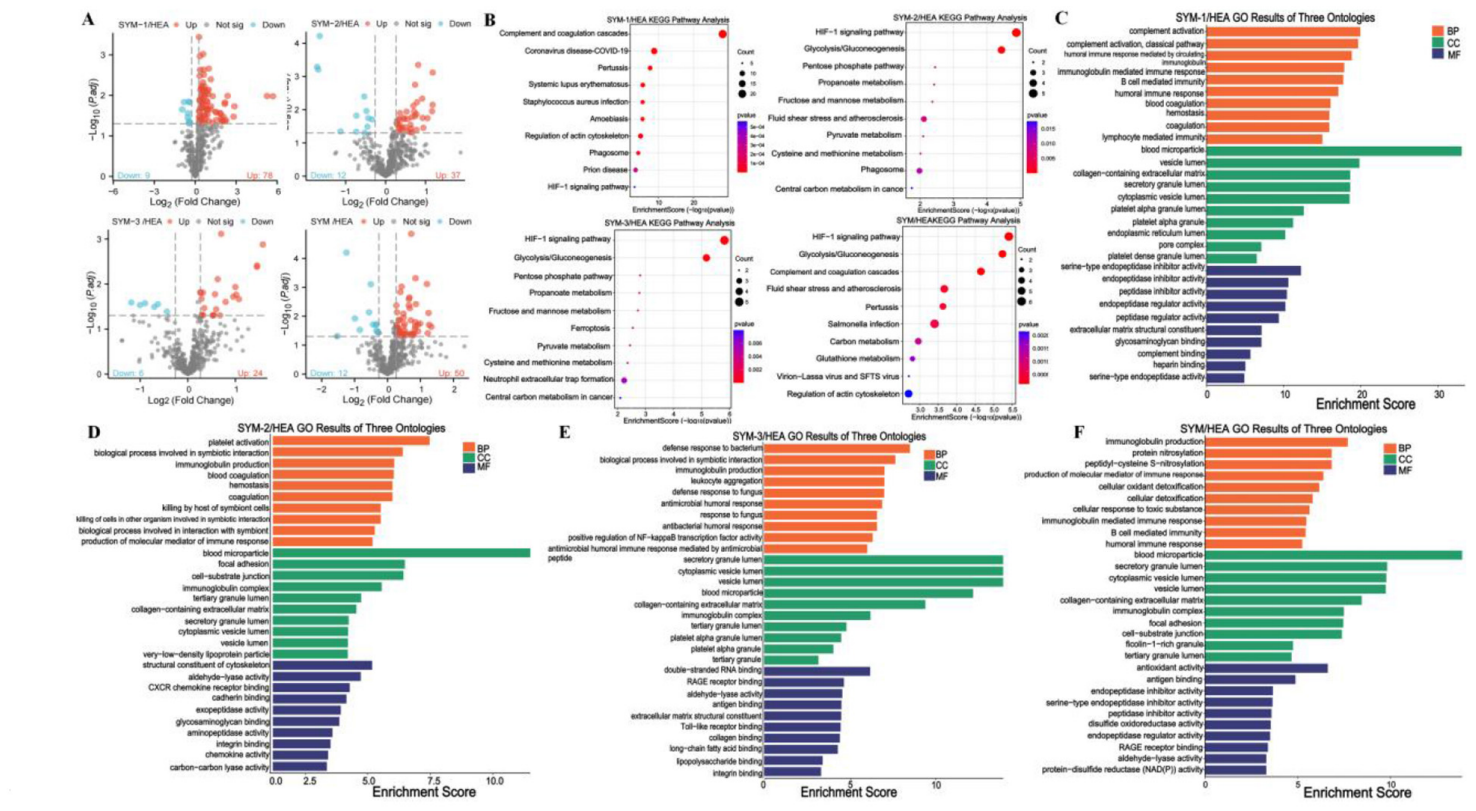
SYM vs. HEA: protein expression and functional enrichment. **(A)** Volcano plots (SYM-1/HEA, SYM-2/HEA, SYM-3/HEA, SYM/HEA). *x*-axis: log_2_ (fold change); *y*-axis: –log_10_ (*P*-value). Dots: proteins (red: upregulated; blue: downregulated; gray: not significant); numbers indicate differential protein counts. **(B)** KEGG pathway enrichment (four SYM/HEA comparisons). *x*-axis: enrichment score [–log_10_ (*P*-value)]; *y*-axis: pathways. Dot size: gene count; color: *P*-value. **(C–F)** GO term enrichment (BP, biological process; CC, cellular component; MF, molecular function) for SYM-1/HEA, SYM-2/HEA, SYM-3/HEA, SYM/HEA. *x*-axis: enrichment score; *y*-axis: terms. Colors distinguish GO categories.

The Cross-sectional serum proteomic analysis of symptomatic COVID-19 subgroups at different clinical stages (incubation SYM-1, acute symptom SYM-2, and recovery SYM-3) relative to HEA revealed distinct dysregulation profiles across subgroups, with the magnitude of molecular alterations varying by disease phase (Figure 3A). The volcano plots showed that SYM-1 triggers upregulation of 78 proteins (dominated by coagulation factors and cytoskeletal mediators) alongside 9 downregulated proteins, such as ADAMTS1. By SYM-2, this signature shifts: 37 proteins (including immune regulators and metabolic enzymes are upregulated, while 12 proteins are downregulated, reflecting the peak inflammatory and functional disruption of acute disease. In the SYM-3 phase, the signature contracts to 24 upregulated proteins and 6 downregulated proteins, with partial normalization of earlier perturbations. The KEGG pathway analysis contextualized the proteomic differences of symptomatic COVID-19 across cross-sectional disease-phase subgroups (Figure 3B). For the SYM-1, top-enriched pathways included complement and coagulation cascades and coronavirus disease-COVID-19, alongside signatures of immune activation, reflecting early pathogen-responsive pathway engagement at greater magnitude than in asymptomatic infection. In the SYM-2 phase, enrichment shifted to metabolic and cellular stress pathways: HIF-1 signaling and glycolysis/gluconeogenesis dominated, consistent with the metabolic collapse and hypoxia linked to severe disease. In the SYM-3 group, enrichment analysis revealed the emergence of the ferroptosis pathway (*FDR* < 0.05), driven by two differentially expressed proteins: downregulated GPX4 and upregulated ACSL4. These proteins are key regulators of ferroptosis, and their co-regulation suggests a pro-ferroptotic cellular environment. While this finding is preliminary due to the small sample size, it is biologically consistent with residual cellular stress and tissue damage observed in the recovery phase of severe COVID-19. Metabolic pathways, including HIF-1 signaling and glycolysis/gluconeogenesis, remained enriched, aligning with ongoing metabolic dysregulation. The combined SYM cohort consolidated these signatures: complement and coagulation cascades and HIF-1 signaling remained top-enriched, with additional representation of Salmonella infection. The GO enrichment analysis delineated the BP, CC, and MF that underpin symptomatic COVID-19's proteomic dynamics (Figures 3C–F). In the SYM-1phase, BP enrichment prioritized complement activation and humoral immune responses, while CC terms focused on vesicle lumens and extracellular matrix components, the MF signatures centered on protease inhibition, consistent with the pro-thrombotic and immune-primed profile of early symptomatic infection (Figures 3C). By the SYM-2 phase, the GO signature expanded to include platelet activation and symbiont interaction processes, with CC terms emphasizing secretory granules and immune complexes. MF signatures shifted toward adhesion and enzyme activity, reflecting widespread cellular and tissue dysregulation. In the SYM-3 phase, BP enrichment reoriented to defense responses and cytokine regulation, CC terms retained vesicle and extracellular compartments, and MF signatures included RNA and receptor binding, marking a transition from acute damage to immune resolution (Figures 3D). The combined SYM cohort consolidated these trends: BP terms highlighted immunoglobulin production and oxidative stress responses, CC terms focused on secretory vesicles and extracellular matrix, and MF signatures persisted for protease inhibition (Figure 3F). Together, these cross-sectional proteomic, pathway, and functional analyses demonstrate that symptomatic COVID-19 subgroups at different clinical stages present distinct molecular profiles: the incubation phase shows prominent activation of coagulation and immune pathways, the acute symptom phase exhibits widespread metabolic and cellular dysregulation, and the recovery phase retains partial damage signatures along with signs of functional normalization. This broad, sustained dysregulation, spanning coagulation, immunity, metabolism, and tissue homeostasis, defines symptomatic infection as a systemically disruptive clinical phenotype, functionally distinct from the targeted, reversible response of asymptomatic COVID-19.

### 3.4 Comparative proteomic analysis identifies distinct core signatures: metabolic constraints in asymptomatic vs. systemic amplification in symptomatic COVID-19

**Figure 4:**
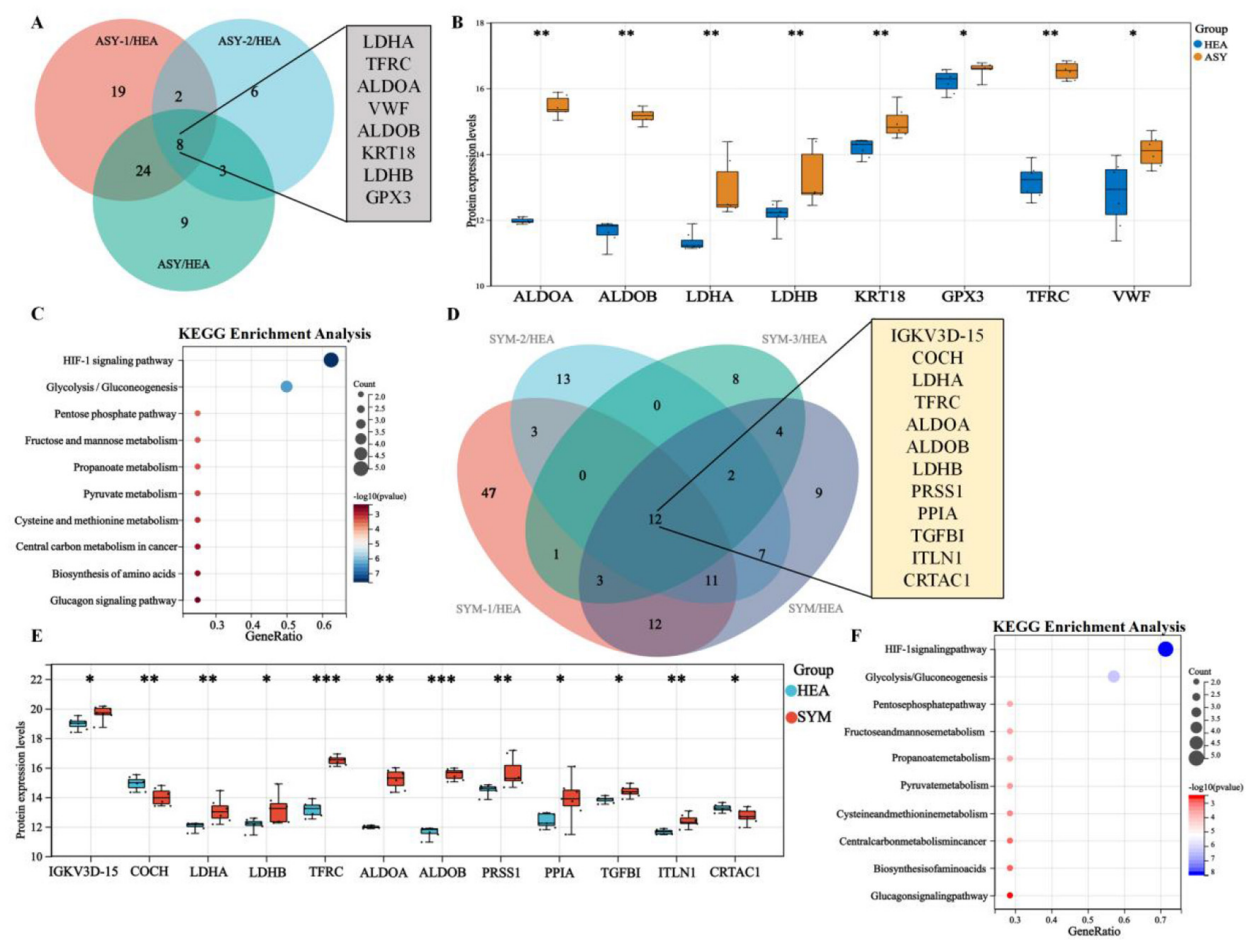
Shared differential proteins and pathway enrichment. **(A)** Venn diagram of differential proteins in ASY-1/HEA, ASY-2/HEA, ASY/HEA; listed proteins are shared candidates. **(B)** Box plots of protein expression (HEA vs. ASY) for key candidates (**P < 0.05, ***P < 0.001). **(C)** KEGG enrichment for ASY/HEA: *x*-axis = GeneRatio; *y*-axis = pathways. Dot size = gene count; color = –log_10_ (*P*-value). **(D)** Venn diagram of differential proteins in SYM-1/HEA, SYM-2/HEA, SYM-3/HEA, SYM/HEA; listed proteins are shared candidates. **(E)** Box plots of protein expression (HEA vs. SYM) for key candidates (*P < 0.05, **P < 0.01, ***P < 0.001). **(F)** KEGG enrichment for SYM/HEA: *x*-axis = GeneRatio; *y*-axis = pathways. Dot size = gene count; color = –log_10_ (*P*-value).

To directly contrast asymptomatic and symptomatic COVID-19 proteomes, the study first mapped shared and unique protein perturbations in asymptomatic cohorts (Figure 4A). This Venn diagram maps the overlap of dysregulated proteins across three asymptomatic COVID-19 cohorts (ASY-1, ASY-2, and combined ASY) relative to HEA. A core set of 8 proteins (LDHA, TFRC, ALDOA, VWF, ALDOB, KRT18, LDHB, GPX3) is dysregulated across all three cohorts, defining the molecular signature of asymptomatic infection. Nineteen proteins are specific to ASY-1, consistent with the acute pathogen-responsive remodeling of active asymptomatic disease. Six proteins are unique to ASY-2, aligning with the metabolic and immune resolution of recovery. Only 2 proteins overlap between ASY-1 and ASY-2, while 3 are shared between ASY-2 and the combined ASY cohort, highlighting the difference in molecular signature breadth between the active infection and recovery subgroups of asymptomatic COVID-19. This study quantitatively analyzed 8 common proteins in the ASY cohort. Results indicate that metabolic enzymes (ALDOA, ALDOB, LDHA, LDHB), coagulation-related proteins (VWF), and intracellular homeostasis-related proteins (KRT18, GPX3, TFRC) are upregulated in the ASY cohort (Figure 4B). KEGG enrichment of these shared asymptomatic proteins prioritizes HIF-1 signaling and glycolytic pathways, aligning with the metabolic focus of asymptomatic recovery (Figure 4C). For symptomatic cohorts, the Venn diagram reveals 12 proteins (IGKV3D-15, COCH, LDHA, TFRC, ALDOA, ALDOB, LDHB, PRSS1, PPIA, TGFBI, ITLN1, CRTAC1) dysregulated across all symptomatic phases (SYM-1, SYM-2, SYM-3), with 47 proteins specific to acute SYM-1, reflecting the amplified dysregulation of active disease (Figure 4D). This study quantitatively analyzed 12 common proteins in the SYM cohort. Results indicate that adaptive immune effectors (IGKV3D-15), metabolic enzymes (LDHA, LDHB, ALDOA, ALDOB), intracellular homeostasis-related proteins (TFRC, PRSS1, PPIA), and tissue remodeling proteins (TGFBI, ITLN1) are upregulated in the SYM cohort, but COCH and CRTAC1 showed lower expression levels in the SYM cohort compared to HEA (Figure 4E). The KEGG enrichment confirmed consistent activation of HIF-1 signaling and glycolytic pathways across both phenotypes, with markedly stronger pathway perturbation in symptomatic groups compared with asymptomatic individuals (Figure 4F). Together, these analyses delineate the molecular phenotypic differences between asymptomatic and symptomatic COVID-19. Both clinical phenotypes shared consistent upregulation direction of core metabolic proteins relative to healthy controls. Asymptomatic infection presented a constrained core signature of 8 proteins (spanning metabolism, coagulation, and intracellular homeostasis), with mild expression alterations across different disease stages and predominant enrichment of metabolic pathways. In contrast, symptomatic disease exhibited a broader amplified signature of 12 proteins, additionally involving adaptive immune and tissue remodeling molecules, with markedly higher expression magnitude of shared metabolic proteins. Of note, these cross-sectional group differences cannot distinguish persistent molecular changes from individual baseline expression variations, due to the independent participant design of our study.

### 3.5 The metabolic core proteins and cross-sectional expression differences across clinical phenotypes of COVID-19

**Figure 5:**
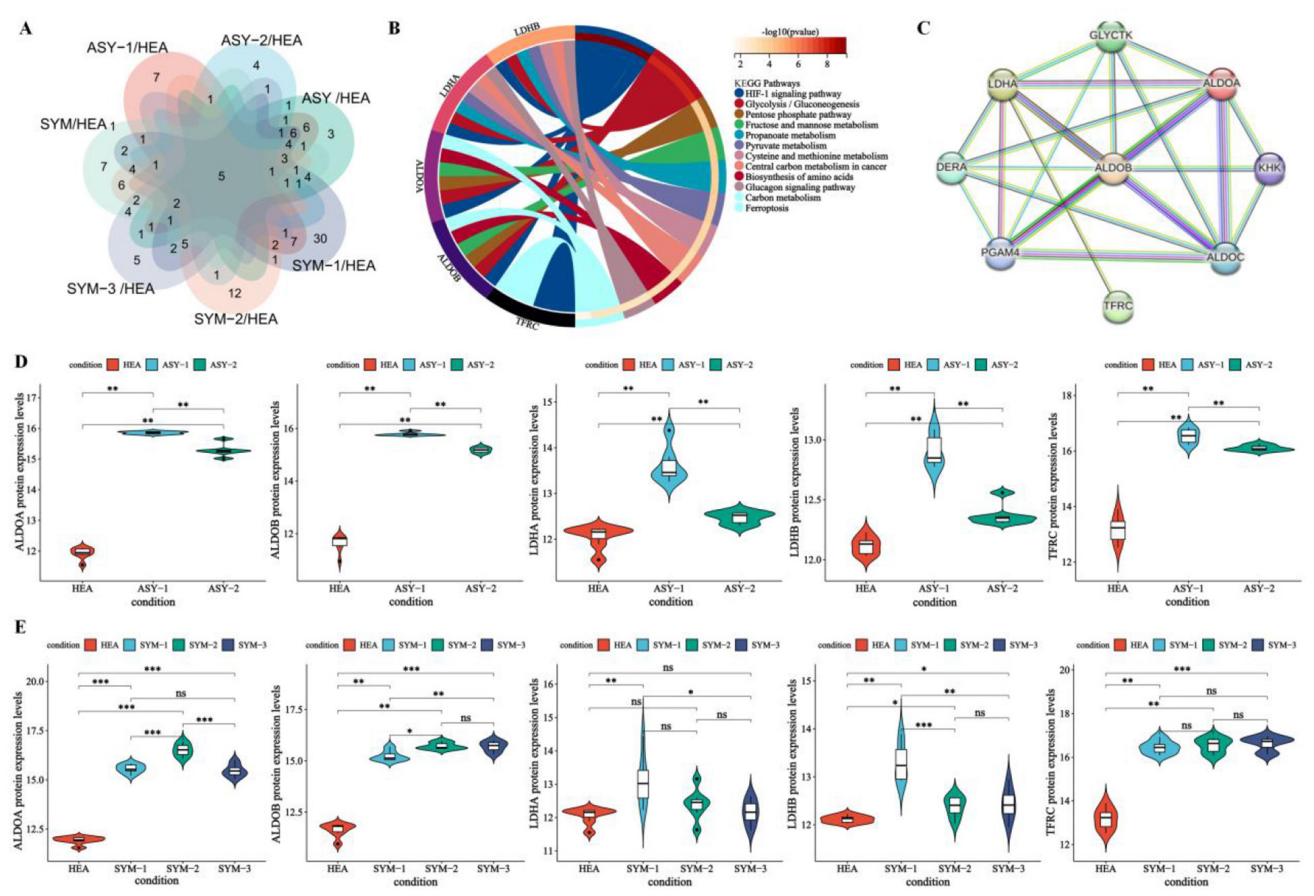
Cross-comparison of differential proteins and networks. **(A)** UpSet plot showing overlapping differential proteins across ASY/SYM vs. HEA subgroups. Numbers indicate shared protein counts. **(B)** Circos plot linking key proteins (outer ring) to enriched KEGG pathways (inner segments). Color intensity: –log_10_ (*P*-value). **(C)** Protein–protein interaction network of glycolysis-related proteins (nodes: proteins; edges: interactions; colors: pathway associations). **(D)** Violin plots of protein expression (ALDOA, ALDOB, LDHA, LDHB, TFRC) in HEA vs. ASY subgroups (**P < 0.01). **(E)** Violin plots of protein expression (ALDOA, ALDOB, LDHA, LDHB, TFRC) in HEA vs. SYM subgroups (**P < 0.01; *P < 0.05; ns, not significant). Violin plots showing cross-sectional expression comparisons across independent participant groups at different clinical stages, not representing longitudinal changes within individual patients.

To identify molecular commonalities between ASY and SYM COVID-19, the study first screened for shared dysregulated proteins across all cohorts. This UpSet plot reveals five conserved proteins (ALDOA, ALDOB, LDHA, LDHB, and TFRC), as the sole set of proteins consistently perturbed in both ASY (ASY-1, ASY-2) and SYM (SYM-1, SYM-2, SYM-3) groups, representing the only functional overlap between the two phenotypes (Figure 5A). The next mapped these five proteins to their enriched biological pathways using a chord diagram, where outer arcs denote proteins, inner arcs denote KEGG pathways, and ribbon width corresponds to enrichment significance [–log(*P*)]. All five proteins cluster tightly with glycolysis/gluconeogenesis and HIF-1 signaling pathways: glycolytic enzymes (ALDOA, ALDOB, LDHA, LDHB) show strong associations with glycolysis, while TFRC (an iron transporter linked to intracellular homeostasis) aligns with HIF-1 signaling (ribbon width ~4), confirming that the shared signature is centered on metabolic and hypoxia-responsive processes (Figure 5B). A PPI network further contextualizes these proteins: ALDOA, ALDOB, LDHA, and LDHB form a tightly connected glycolytic module anchored by GLYCTK, while TFRC acts as a peripheral node linking this metabolic core to intracellular iron homeostasis (Figure 5C). This network structure is conserved across both phenotypes, though SYM-specific proteins expand the module to include immune effectors, reinforcing metabolism as the foundational shared pathway. To characterize group-level expression differences of these five proteins across clinical stages, we generated violin plots for comparison (Figures 5D, E). In asymptomatic cohorts, ALDOA, ALDOB, LDHA, LDHB and TFRC displayed significantly higher expression in the ASY-1 group compared with the ASY-2 group (*P* < 0.05), where expression levels were close to those of healthy controls (HEA; Figure 5D). Notably, ASY-1 and ASY-2 consist of independent participants, so these differences reflect cross-sectional variation between distinct subgroups rather than intra-individual changes over infection progression. For symptomatic cohorts covering SYM-1, SYM-2 and SYM-3, all five core proteins were significantly upregulated relative to HEA (Figure 5E). ALDOA and ALDOB showed high expression in the SYM-1 group, and elevated expression was observed across all symptomatic subgroups. LDHA presented approximately 3-fold higher expression in SYM-1, and high expression was maintained across SYM-2 and SYM-3. LDHB and TFRC exhibited similar expression patterns across all symptomatic stages. Overall, the expression magnitude of these core proteins in symptomatic groups was substantially higher than that in asymptomatic groups.

Collectively, these analyses identify five conserved metabolic and homeostasis-related proteins as the key molecular overlap between asymptomatic and symptomatic COVID-19. Both clinical phenotypes exhibit activation of glycolysis/gluconeogenesis and HIF-1 signaling pathways. The major distinction between the two phenotypes lies in the expression magnitude across cross-sectional subgroups. The active infection subgroup of asymptomatic cases shows higher protein abundance than the recovery subgroup, while all symptomatic disease-phase subgroups exhibit consistently high expression of these core metabolic proteins relative to healthy controls. These population-level differences distinguish the molecular profiles of asymptomatic and symptomatic COVID-19.

### 3.6 SMR analysis links ALDOB cis-eQTL variation to COVID-19 susceptibility

**Figure 6:**
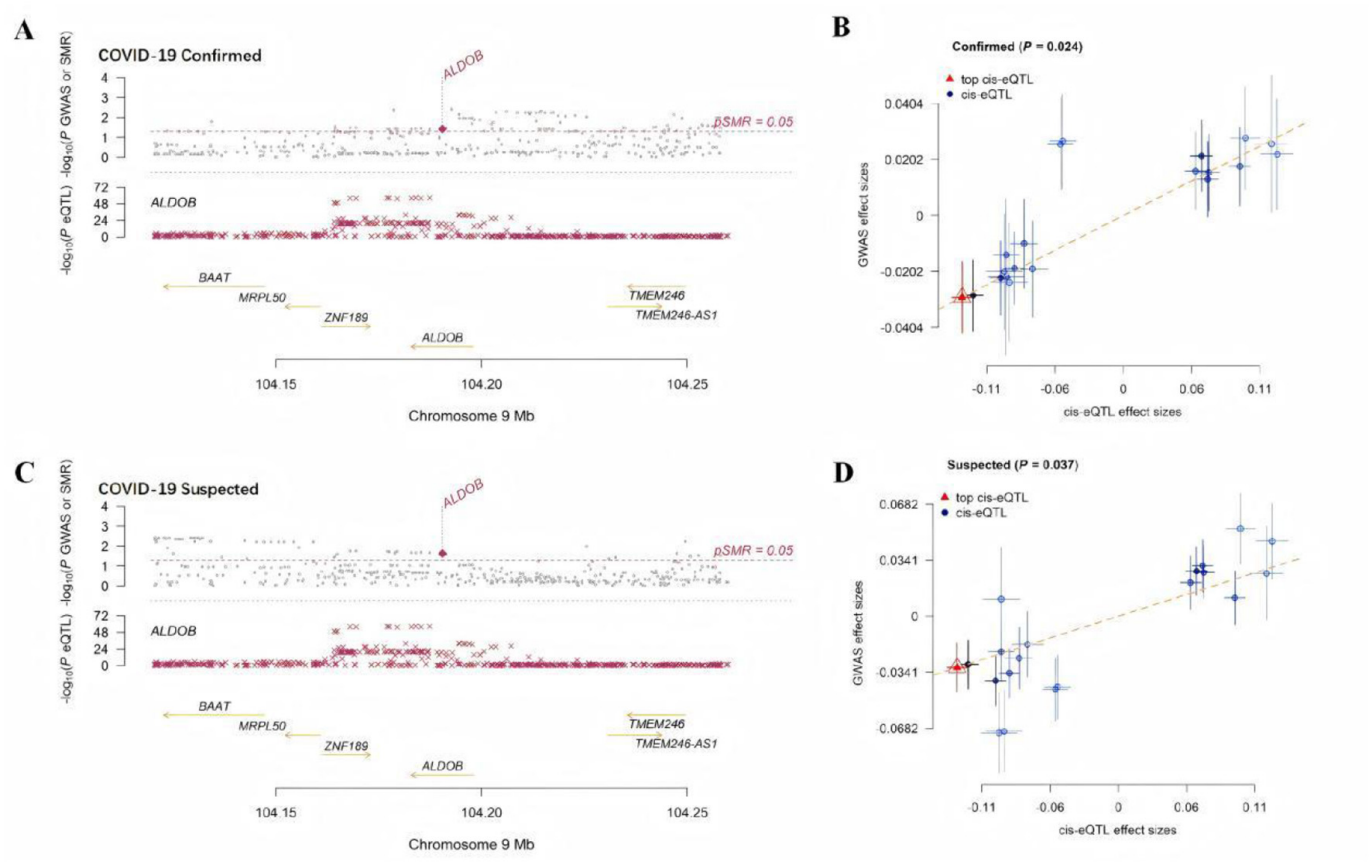
ALDOB cis-eQTL and COVID-19 GWAS association. **(A)** COVID-19 confirmed: regional GWAS [top, –log_10_ (*P*)] and cis-eQTL [bottom, –log_10_ (*P*)] plots for chromosome 9 (104.15–104.25 Mb). Red dot: ALDOB; dashed line: *P* = 0.05. Genes are annotated. **(B)** Scatter plot of cis-eQTL vs. GWAS effect sizes (confirmed cohort, *P* = 0.024). Red dot: top ALDOB cis-eQTL; blue dots: other cis-eQTLs; dashed line: trend. **(C)** COVID-19 suspected: regional GWAS and cis-eQTL plots (chromosome 9, same region as A). Red dot: ALDOB; dashed line: *P* = 0.05. **(D)** Scatter plot of cis-eQTL vs. GWAS effect sizes (suspected cohort, *P* = 0.037). Red dot: top ALDOB cis-eQTL; blue dots: other cis-eQTLs; dashed line: trend.

To investigate causal relationships between these metabolic proteins and COVID-19, we performed SMR analysis, evaluating cis-eQTLs and cis-pQTLs as exposures for two COVID-19 outcomes (Confirmed, Suspected). For ALDOB, cis-eQTL variation was significantly associated with both COVID-19 confirmed (*P* = 0.024) and suspected (*P* = 0.037) outcomes, with OR of 1.249 (95% *CI*: 1.030–1.513) and 1.265 (95% *CI*: 1.014–1.577), respectively. HEIDI tests (*P* = 0.743 for Confirmed; *P* = 0.249 for Suspected) ruled out horizontal pleiotropy, supporting a causal effect of ALDOB expression on COVID-19 susceptibility. In contrast, ALDOB cis-pQTL variation showed no significant association with either outcome (*P* > 0.5 for both), indicating the causal signal is driven by transcript (rather than protein) regulation (Table 1). For the remaining proteins (LDHA, LDHB, TFRC), no significant associations were observed between cis-eQTL/cis-pQTL variation and COVID-19 outcomes (all *P* > 0.05), among these, ALDOA was excluded from the analysis due to the lack of strongly associated SNPs. Regional association plots visualize the ALDOB cis-eQTL signal at chromosome 9q34, with the lead variant (rs4577) aligning with ALDOB expression (Figures 6A, C); scatter plots confirm the positive correlation between ALDOB cis-eQTL effect size and COVID-19 risk effect size, consistent with the SMR results (Figures 6B, D). These findings identify ALDOB cis-eQTL-mediated expression as a candidate factor associated with COVID-19 susceptibility, while the other conserved metabolic proteins in the shared core do not exhibit detectable associations. It should be noted that this association was identified using European-derived eQTL data, and its replicability in East Asian populations remains to be verified. These results highlight ALDOB as a potential key functional node within the metabolic pathway that may contribute to COVID-19 phenotypic differences.

**Table 1:** Results of the SMR analysis.

Expousre	Type	Outcome	*P*	HEIDI *P*	*OR* (95% *CI*)
ALDOB	cis-eQTL	COVID-19 confirmed	**0.024**	**0.743**	1.249 (1.030, 1.513)
ALDOB	cis-eQTL	COVID-19 suspected	**0.037**	**0.249**	1.265 (1.014, 1.577)
ALDOB	cis-pQTL	COVID-19 confirmed	0.670	0.870	0.983 (0.910, 1.062)
ALDOB	cis-pQTL	COVID-19 suspected	0.512	0.921	0.971 (0.888, 1.061)
LDHA	cis-eQTL	COVID-19 confirmed	0.404	0.495	1.066 (0.918, 1.237)
LDHA	cis-eQTL	COVID-19 suspected	0.573	0.547	0.952 (0.803, 1.129)
LDHA	cis-pQTL	COVID-19 confirmed	0.317	0.890	1.281 (0.788, 2.082)
LDHA	cis-pQTL	COVID-19 suspected	0.989	0.390	1.004 (0.590, 1.709)
LDHB	cis-eQTL	COVID-19 confirmed	0.671	0.088	1.053 (0.830, 1.335)
LDHB	cis-eQTL	COVID-19 suspected	0.977	0.412	1.004 (0.769, 1.310)
LDHB	cis-pQTL	COVID-19 confirmed	0.899	0.949	1.023 (0.723, 1.448)
LDHB	cis-pQTL	COVID-19 suspected	0.794	0.819	1.056 (0.702, 1.589)
TFRC	cis-eQTL	COVID-19 confirmed	0.834	0.374	1.011 (0.911, 1.123)
TFRC	cis-eQTL	COVID-19 suspected	0.142	0.247	0.914 (0.811, 1.030)
TFRC	cis-pQTL	COVID-19 confirmed	0.927	0.882	0.983 (0.682, 1.416)
TFRC	cis-pQTL	COVID-19 suspected	0.686	0.170	1.090 (0.716, 1.660)

Bold values indicate statistically significant *P* values (*P* < 0.05).

### 3.7 Specific circulating glycerol, not global glucose homeostasis traits, is a causal risk factor for confirmed COVID-19

**Figure 7:**
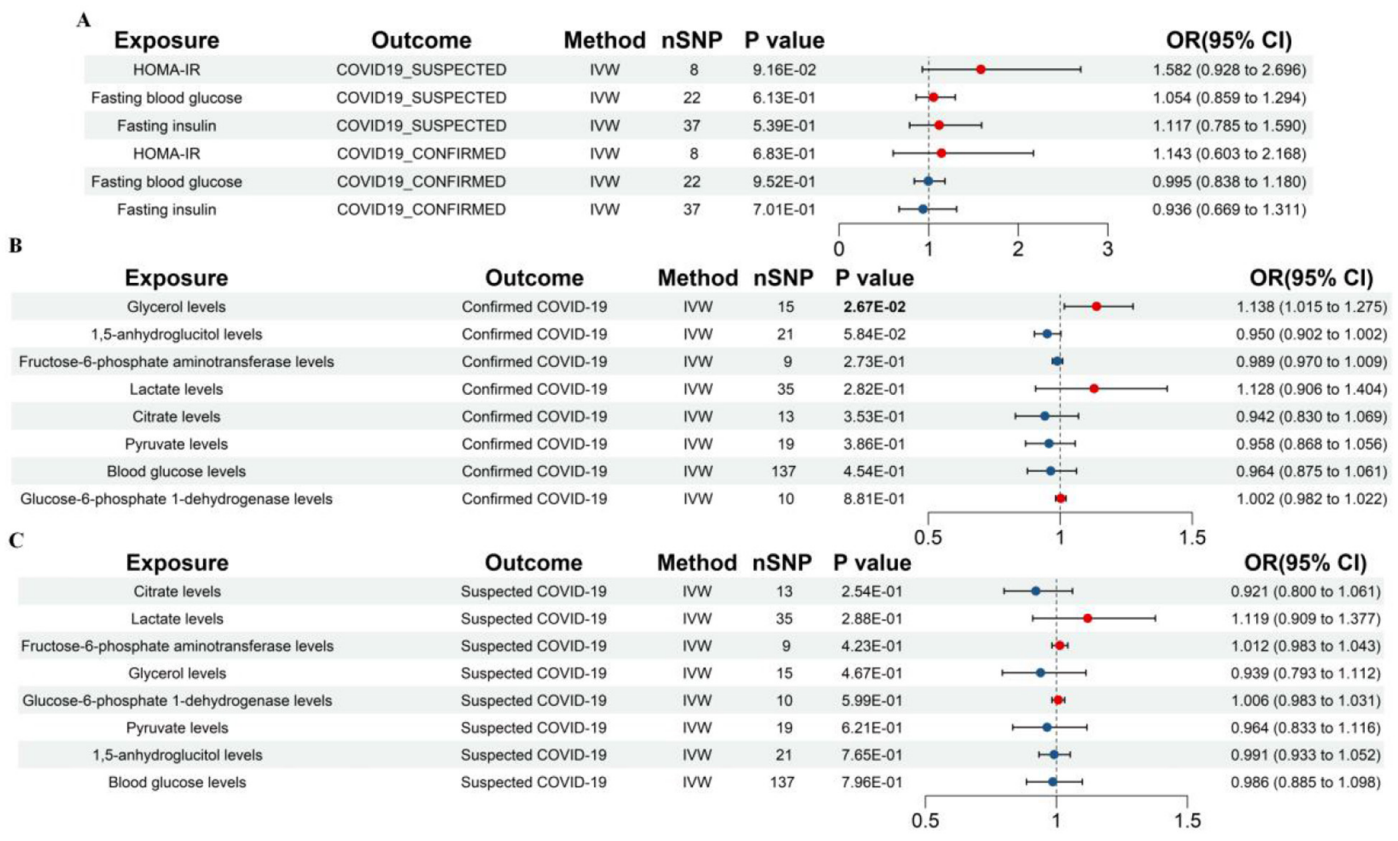
Mendelian randomization of metabolic traits and COVID-19 risk. **(A)** Inverse-IVW MR results for insulin/glucose-related exposures (HOMA-IR, fasting glucose/insulin) vs. COVID-19 (suspected/confirmed). Table columns: exposure, outcome, method, SNP count (nSNP), *P*-value, and odds ratio (OR, 95% CI). Forest plot: dots = OR; bars = 95% CI (red/blue = different exposures). HOMA-IR shows a suggestive link to suspected COVID-19 (*OR* = 1.582, *P* = 0.0916). **(B)** IVW MR results for metabolic metabolite exposures vs. Confirmed COVID-19. Glycerol levels associate with elevated risk (*OR* = 1.138, *P* = 0.0267); other metabolites show no significant associations. **(C)** IVW MR results for metabolic metabolite exposures vs. suspected COVID-19. No metabolites exhibit statistically significant associations with suspected COVID-19 (all *P* > 0.05).

Preliminary results indicate that biological enrichment analysis of differentially expressed proteins in both asymptomatic carriers and confirmed COVID-19 samples consistently reveals associations with glucose metabolism biological functions. Therefore, to determine whether systemic glucose homeostasis traits (HOMA-IR, fasting blood glucose, fasting insulin) contribute to COVID-19 risk, this study performed bidirectional MR. The MR analysis assessed the causal association between three glucose metabolism indicators and COVID-19. HOMA-IR (*OR* = 1.58, 95% *CI*: 0.93–2.70, *P* = 0.09), fasting blood glucose (*OR* = 1.05, 95% *CI*: 0.86–1.29, *P* = 0.61), and fasting insulin (*OR* = 1.12, 95% *CI*: 0.78–1.59, *P* = 0.54) showed no significant association with suspected COVID-19. Similarly, HOMA-IR (*OR* = 1.14, 95% *CI*: 0.60–2.17, *P* = 0.68), fasting glucose (*OR* = 0.99, 95% *CI*: 0.84–1.18, *P* = 0.95), and fasting insulin (*OR* = 0.94, 95% *CI*: 0.67–1.31, *P* = 0.70) were not significantly associated with confirmed COVID-19 (Figure 7A). Similarly, MR analysis results did not reveal any significant inverse causal relationship between the three glucose metabolism indicators and COVID-19. All results passed tests for heterogeneity and multi-effectiveness (*P* > 0.05). In summary, MR analysis findings did not reveal a significant causal association between the three glucose metabolism indicators and COVID-19. Although no significant causal association was found between baseline glucose metabolism indicators (HOMA-IR, fasting blood glucose, fasting insulin) and COVID-19 risk, the study further expanded its analysis to be narrowed to specific cyclic metabolites within the glucose metabolic pathway to investigate whether targeting metabolic intermediates constitutes the basis for COVID-19 disease risk, based on the sustained significance of glucose metabolism pathways in the preliminary protein enrichment analysis. The MR analysis assessed the causal association between 8 glucose metabolism indicators and COVID-19. MR analysis evaluated the causal association between multiple circulating metabolites and COVID-19. Results showed elevated glycerol levels were a risk factor for confirmed COVID-19 (*OR* = 1.14 95% *CI*: 1.01–1.28, *P* = 0.027; Figure 7B). Other metabolites, including fructose-6-phosphate aminotransferase, lactate, citrate, pyruvate, blood glucose, and glucose-6-phosphate dehydrogenase 1 levels, showed no significant association with confirmed COVID-19 (*P* > 0.05). Furthermore, no significant causal association was found between any analyzed metabolite levels and suspected COVID-19 (*P* > 0.05; Figure 7C). All results passed heterogeneity tests (*P* > 0.05) and multiplicity tests (*P* > 0.05, and reverse Mendelian randomization revealed no significant reverse causality (*P* > 0.05). In summary, MR analysis identified elevated glycerol levels as a risk factor for confirmed COVID-19. These findings suggest that while overall glucose homeostasis indicators may not directly influence susceptibility to COVID-19, specific metabolites, particularly glycerol, may play a significant role in the pathogenesis of the disease. Comprehensive analysis indicates that the role of glucose metabolic pathways in COVID-19 may not be reflected through traditional homeostasis indicators, but rather through abnormalities in specific metabolic processes.

## 4 Discussion

The comprehensive integration of serum proteomic profiling, functional genomics, and causal inference analysis presented in this study identifies molecular profiles associated with the clinical heterogeneity of COVID-19. These data collectively suggest a model wherein asymptomatic and symptomatic infections are not positioned along a linear spectrum of disease severity, but represent distinct biological phenotypes with divergent molecular profiles across tested groups. All samples from different clinical stages were collected from independent individuals, rather than from a single cohort with serial longitudinal sampling. The asymptomatic group exhibits a focused molecular profile dominated by coagulation and immediate innate immune responses. Across cross-sectional groups defined by different disease phases, protein expression patterns differ notably between subgroups. In the subgroup corresponding to early infection, this immune-coagulation signature is prominent, while subgroups representing later infection phases show distinct molecular features with elevated activity in metabolic pathways. In comparison, symptomatic groups present broadly altered molecular profiles across all tested stages, with combined activation of coagulation, immunity, metabolism and tissue remodeling-related molecules. The molecular divergence between these two clinical phenotypes lies in both the range of affected molecules and the overall expression levels of key proteins. Crucially, the study identifies a conserved core of five metabolic and hypoxia-responsive proteins (ALDOA, ALDOB, LDHA, LDHB, TFRC) as the sole nexus connecting these disparate clinical outcomes. Through exploratory MR analyses, the study further identified genetic variation influencing ALDOB expression and elevated circulating glycerol levels as suggestive candidate correlates of COVID-19 susceptibility, highlighting specific nodes within the broader metabolic dysregulation for follow-up investigation.

The predominant upregulation of fibrinogen subunits and the marked enrichment of the complement and coagulation cascades are consistent with the critical role of immune-thrombosis in pathogen containment (Engelmann and Massberg, 2013; Skendros et al., 2020). The molecular profile of asymptomatic groups shares features with the immune-coagulation response observed in symptomatic groups, but lacks strong signals linked to extensive complement activation, inflammatory cascades and epithelial injury markers such as S100A8/A9 (Shen et al., 2020; Laing et al., 2020). Cross-sectional comparison between two asymptomatic subgroups shows distinct pathway enrichment: one subgroup is characterized by prominent complement and coagulation activity, whereas the other shows stronger enrichment of glycolysis and gluconeogenesis pathways. This group-level difference indicates that effective pathogen control can be achieved without extensive systemic tissue injury. This apparent contraction of the dysregulated proteome and functional shift toward glycolysis and gluconeogenesis pathways in the ASY-2 phase suggests that efficient viral clearance may occur without triggering a maladaptive systemic inflammatory cascade (Galbraith et al., 2022). The prominent coagulation signature, even in the absence of symptoms, underscores the profound pro-thrombotic nature of SARS-CoV-2 infection from its earliest stages (Ackermann et al., 2020). Meanwhile, markers of severe epithelial and endothelial damage are largely absent in asymptomatic individuals, further distinguishing the two clinical phenotypes (Abers et al., 2021).

Symptomatic COVID-19 groups show extensive molecular alterations across all sampled stages. Cross-sectional comparison among symptomatic subgroups reveals differing functional pathway enrichment: one subgroup features dominant coagulation and immune signals, while others show prominent metabolic dysregulation. Strong upregulation of glycolytic enzymes (ALDOA, ALDOB, LDHA, LDHB) and consistent enrichment of HIF-1 signaling across symptomatic groups are typical features of Warburg-like metabolic reprogramming, which is commonly observed in severe infectious diseases and critical illnesses (Cheng et al., 2014; Codo et al., 2020). Such metabolic changes may meet the energy demands of hyperactivated immune cells and correlate with tissue hypoxia, which may contribute to aggravated inflammation and tissue damage (O'Neill et al., 2016), though this mechanism remains speculative. One symptomatic subgroup shows notable enrichment of the ferroptosis pathway, which correlates with disturbed iron metabolism and oxidative damage, and is thought to be involved in long-term organ complications after COVID-19 infection (Amaral and Namasivayam, 2021). The broad GO terms encompassing platelet activation, symbiont interaction, and extracellular matrix organization in the symptomatic subgroup may reflect systemic thrombosis and tissue fibrosis that characterizes severe disease (Gu et al., 2021; Zuo et al., 2021), based on cross-sectional observations. Comparative analysis across all groups demonstrates that asymptomatic and symptomatic COVID-19 share altered activity in core metabolic and hypoxia-responsive pathways, while differing substantially in the overall scale of molecular changes and protein expression levels. The co-upregulation of five core proteins (ALDOA, ALDOB, LDHA, LDHB, TFRC) indicates that SARS-CoV-2 enriches central carbon metabolism and the hypoxic response regardless of outcome, suggesting that SARS-CoV-2 universally triggers a metabolic stress response in the host (Chen et al., 2023). The main difference between phenotypes lies in the magnitude of such responses. Symptomatic groups present higher expression of these core metabolic proteins and additional activation of immunoglobulin and tissue remodeling-related molecules, reflecting impaired physiological homeostasis (Filbin et al., 2021). This suggests clinical severity is determined not by the activation of unique pathways, but by the failure to resolve a common foundational response.

The SMR analysis providing preliminary evidence that ALDOB cis-eQTL variants may be associated with increased susceptibility to COVID-19 is a pivotal preliminary finding. ALDOB, a fructose-1,6-bisphosphate aldolase, is central to glycolysis and gluconeogenesis (Li et al., 2020). The study preliminarily suggests that genetically determined higher ALDOB expression may potentially predispose individuals to a more robust or dysregulated metabolic response upon infection, thereby possibly increasing the risk of progressing to symptomatic disease. This aligns with studies linking metabolic gene variants to infectious disease outcomes and highlights host metabolic genotype as a previously underappreciated determinant of COVID-19 severity (Lee et al., 2024). This finding proposes that inter-individual genetic variation in the regulation of metabolic genes can predispose to differential clinical outcomes upon viral challenge (Wang et al., 2020; Fricke-Galindo and Falfan-Valencia, 2021). The lack of association with ALDOB pQTLs implies the causal mechanism operates at the transcriptional or translational regulation level, meriting further investigation. Our analyses revealed that genetic variation associated with ALDOB mRNA expression was linked to COVID-19 risk, whereas no significant association was detected between genetically predicted circulating ALDOB protein levels and disease susceptibility. This seemingly contradictory result is consistent with the intrinsic biological features of ALDOB. As a metabolism- and signal-related hub protein with strong tissue specificity, ALDOB expression is tightly modulated by dietary factors including fructose (Chen et al., 2022). More importantly, its core biological functions are executed through various post-translational modifications such as phosphorylation and acetylation, as well as direct physical interactions with critical molecules including IR, G6PD and KAT2A (Wang et al., 2026; Liu et al., 2021; Chang et al., 2018). Such functional regulation is not entirely dependent on changes in total protein abundance. Furthermore, the stability of ALDOB protein is actively regulated via the lysosomal pathway (Susan and Dunn, 2001). Collectively, our findings suggest that ALDOB may influence the pathophysiology of COVID-19 through sophisticated post-translational regulation and protein interaction networks, rather than simply via altered circulating protein levels.

Extending this metabolic theme, our MR analysis suggests circulating glycerol may be a potential causal risk factor for confirmed COVID-19, though the MR signal is modest and this finding should be interpreted cautiously. Elevated glycerol may indicate increased lipid turnover and energy demand, potentially fueling the hypermetabolic state of severe COVID-19 (Thomas et al., 2020). Its identification as a causal factor suggests that a pre-existing state of altered substrate metabolism may predispose to severe infection. Notably, traditional measures of systemic glucose homeostasis such as HOMA-IR showed no causal effect, indicating that susceptibility is linked to specific dynamic metabolic perturbations rather than chronic abnormal glucose metabolism (Danlos et al., 2021; Thomas et al., 2020). This is corroborated by metabolomic studies identifying distinct lipid and energy metabolism disturbances in patients with severe outcomes (Wu et al., 2020).

Additionally, this study has certain limitations. First, ancestry mismatch between the proteomic cohort and the genetic datasets represents a major limitation of this study. The serum proteomic profiling was performed in a Chinese population, while all cis-eQTL, cis-pQTL, and COVID-19 GWAS datasets used for causal inference were generated from European ancestry populations. Accumulating evidence has demonstrated that cis-regulatory effects on gene expression and protein abundance are strongly ancestry-specific, with only 30–50% of eQTL/pQTL effects shared between European and East Asian populations. Differences in allele frequencies, linkage disequilibrium structures, and regulatory element polymorphisms may attenuate the effect of genetic instruments, reduce statistical power, and introduce bias in effect size estimation. This population heterogeneity in transcript-protein coupling may also partially explain the discrepancy between significant cis-eQTL associations and null cis-pQTL associations for ALDOB, as European-derived pQTLs may poorly proxy circulating protein levels in Chinese individuals. Furthermore, the causal effect estimates derived from European GWAS data cannot be directly extrapolated to East Asian populations, given differences in host genetic background, baseline metabolic profiles, and epidemiological context. All causal inference findings in this study are considered preliminary exploratory results, and independent validation in large-scale East Asian population cohorts is essential before drawing definitive conclusions. Second, serum proteomics data reflect systemic protein expression changes without precise localization to specific cellular or tissue origins. Third, as this study adopted a cross-sectional design with independent participants enrolled in each clinical stage, we are unable to differentiate long-term persistent molecular alterations from inherent baseline expression differences between individuals with distinct COVID-19 clinical outcomes. Longitudinal follow-up cohorts are required to verify the temporal dynamic changes of these core protein signatures. Fourth, this study adopted a cross-sectional design with independent participants recruited for each clinical stage of COVID-19. Consequently, we cannot distinguish true temporal expression changes within a single patient from inherent baseline expression differences among different individuals. Alternative explanations, such as varying infection severity or intrinsic molecular heterogeneity across patient subgroups, cannot be fully excluded without longitudinal follow-up samples. Future longitudinal cohort studies will help verify the dynamic changes of these core protein signatures during infection and recovery. The relatively small sample size of this study may limit the statistical power and generalizability of the observed findings. Furthermore, this was a retrospective study in which samples from different clinical stages were derived from different individuals rather than a single cohort with serial follow-up, which limited the delineation of longitudinal changes during disease progression. Notably, our SMR analysis adopted a nominal *P* < 0.05 threshold without FDR correction for multiple testing of 4,907 proteins, rendering associations such as ALDOB preliminary. The suggestive associations between ALDOB cis-eQTL variation, circulating glycerol and COVID-19 susceptibility may represent false positives due to unadjusted multiple testing. These preliminary results urgently require independent replication and validation in larger cohorts. Future studies require validation across multi-ethnic cohorts and integration with technologies such as single-cell sequencing to further elucidate the cellular origins of these protein alterations.

## 5 Conclusion

Based on cross-sectional comparisons of different clinical-stage subgroups (each consisting of independent individuals), this study proposes a working hypothesis that the clinical outcome of SARS-CoV-2 infection may be associated with the magnitude of the host's metabolic-immune response. Asymptomatic infection is associated with a constrained molecular profile, while symptomatic disease is associated with broadly amplified metabolic and immune dysregulation. The exploratory findings linking ALDOB expression and glycerol metabolism to COVID-19 susceptibility highlight candidate metabolic nodes for further investigation; independent validation is required to clarify their causal roles and translational potential.

## Data Availability

The original contributions presented in the study are publicly available. This data can be found here: The mass spectrometry proteomics data generated in this study have been deposited to the ProteomeXchange Consortium via the PRIDE partner repository with the dataset identifier PXD080937. The dataset is available at https://proteomecentral.proteomexchange.org.
