# Supplementary material for: Metabolic reprogramming is associated with symptomatic COVID-19: a serum proteomics and causal inference study identifying ALDOB and glycerol as candidate metabolic correlates
Source: Front Microbiol. 2026 Jul 24;17:1852103. doi: 10.3389/fmicb.2026.1852103 (PMC13447426; doi:10.3389/fmicb.2026.1852103)
Supplement: Supplementary file 1 [file Table_1.docx]

**Supplementary Table 1: Basic information of research subjects**

|  | HEA | SYM-1 | SYM-2 | SYM-3 | ASY-1 | ASY-2 | statistical value | *P* value |
| --- | --- | --- | --- | --- | --- | --- | --- | --- |
| Gender  (Male/Female) | 3/4 | 4/3 | 5/3 | 3/3 | 3/3 | 3/3 | -- | *P* =0.995 |
| Age  （‾*χ*±S ） | 32.57±1.72 | 32.57±1.27 | 32.75±1.28 | 32.83±1.17 | 31.86±1.46 | 31.86±1.46 | *F* =0.411 | *P* =0.838 |
